# Population-Based Study of Drug-Resistant Epilepsy Before Age Two: Predominance of Developmental and Epileptic Encephalopathies

**DOI:** 10.3390/neurolint18050076

**Published:** 2026-04-22

**Authors:** Stella Lilles, Klari Heidmets, Kaisa Teele Oja, Karit Reinson, Laura Roht, Sander Pajusalu, Monica H. Wojcik, Katrin Õunap, Inga Talvik

**Affiliations:** 1 Department of Pediatrics, Institute of Clinical Medicine, University of Tartu, 50406 Tartu, Estonia; klari.heidmets@kliinikum.ee (K.H.); inga.talvik@lastehaigla.ee (I.T.); 2 Children’s Clinic, Tartu University Hospital, European Reference Network EpiCARE, 50406 Tartu, Estonia; 3 Department of Genetics and Personalized Medicine, Institute of Clinical Medicine, University of Tartu, 50406 Tartu, Estonia; kaisateele.oja@kliinikum.ee (K.T.O.); karit.reinson@kliinikum.ee (K.R.); sander.pajusalu@kliinikum.ee (S.P.); katrin.ounap@kliinikum.ee (K.Õ.); 4 Genetics and Personalized Medicine Clinic, Tartu University Hospital, 50406 Tartu, Estonia; laura.roht@kliinikum.ee; 5 Divisions of Newborn Medicine and Genetics and Genomics, Department of Pediatrics, Boston Children’s Hospital, Harvard Medical School, Boston, MA 02115, USA; monica.wojcik@childrens.harvard.edu; 6 Manton Center for Orphan Disease Research, Division of Genetics and Genomics, Department of Pediatrics, Boston Children’s Hospital, Harvard Medical School, Boston, MA 02115, USA; 7 Broad Center for Mendelian Genomics, Broad Institute of MIT and Harvard, Cambridge, MA 02142, USA; 8 Tallinn Children’s Hospital, European Reference Network EpiCARE, 13419 Tallinn, Estonia

**Keywords:** early-onset epilepsy, drug-resistant epilepsy, developmental and epileptic encephalopathies, epilepsy syndromes, genetic epilepsy, neuroimaging, epilepsy etiology

## Abstract

**Background/Objectives:** Early-onset epilepsy is associated with a high risk of developing drug-resistant epilepsy (DRE), often manifesting as developmental and epileptic encephalopathies (DEEs). This study aimed to characterize the incidence, syndromes, comorbidities, and etiology of early-onset DRE in Estonia. **Methods:** This study is a continuation of our earlier nationwide, population-based investigation and included all children with early-onset epilepsy (seizure onset before two years) who developed drug resistance in Estonia between 2013 and 2017 (*n* = 37). Cases were identified at the country’s only two pediatric neurology departments, ensuring nationwide coverage. Clinical data, electroencephalography, neuroimaging, genetic investigations (chromosomal microarray, single-gene tests, gene panels, exome/genome sequencing), and etiology were analyzed overall and by epilepsy type or syndrome. **Results:** A total of 37 children with early-onset DRE were included. The incidence of early-onset DRE was 26.5 per 100,000 person-years, peaking in the first year of life (36.1). Drug resistance developed in 43% within six months and 65% within one year. DEEs accounted for 76% of cases, most commonly infantile epileptic spasms syndrome (IESS/West syndrome, 35%). Structural abnormalities were observed in 49% of cases (50% of DEEs), most commonly congenital brain malformations (22%). Pathogenic genetic findings were identified in 41% overall (43% of DEEs). The etiology was established in 78% of children with DRE. Among DEEs, it was found in all Dravet syndrome patients (100%) and 62% of those with IESS/West syndrome. Global developmental delay/intellectual disability occurred in 86%, and motor impairment in 46%. **Conclusions:** Early-onset DRE, often presenting as DEE, has high incidence, progresses rapidly to drug resistance, and causes substantial comorbidities.

## 1. Introduction

There is limited data on drug-resistant epilepsy (DRE) in children diagnosed before the age of two years. Previous studies have largely addressed early-onset epilepsy more broadly or DEEs separately in that age group, leaving an important knowledge gap that our study aims to address.

According to the International League Against Epilepsy (ILAE), DRE is defined as the failure of two appropriately selected and well-tolerated anti-seizure medication (ASM) regimens—used either as monotherapy or in combination—to achieve sustained seizure freedom [1]. DRE represents a heterogeneous group of epilepsies, with both focal and generalized forms, and may include refractory epilepsy syndromes such as Dravet syndrome, West syndrome, Lennox–Gastaut syndrome, or Rasmussen’s encephalitis [2,3,4]. The incidence rate of epilepsy is highest in the first year of life (149 per 100,000 person-years) and declines thereafter [5].

During the first two years of life, the brain undergoes rapid, complex, and dynamic development that plays an essential role in cognitive function [6]. Early-onset epilepsy is associated with adverse neurodevelopmental outcomes [7]. In a Swedish study involving children with infantile-onset epilepsy, intellectual disability (ID) was diagnosed in 49%, autism spectrum disorder in 11%, and cerebral palsy in 24% of the children [8]. Jonsson et al. reported that in a prospective cohort of 60 infants with epilepsy onset before 12 months, 37% developed DRE and nearly half had global developmental delay (GDD) by 24 months [9]. Similarly, Berg et al. showed that in 775 children with epilepsy onset before three years, 35% developed DRE, 23% had developmental impairment, and nearly all deaths occurred in infants with epilepsy onset before 12 months, with younger age and early developmental delay predicting poorer outcomes [10].

Early-onset DRE often presents within the spectrum of developmental and epileptic encephalopathies (DEEs), in which early, frequent, drug-resistant seizures and developmental delay or regression are both core features. DEE is a condition in which underlying pathology and ongoing epileptic activity together cause more severe cognitive and behavioral impairments than expected from the pathology alone [11]. Many well-defined epilepsy syndromes, including Dravet syndrome, infantile epileptic spasms syndrome (IESS, including West syndrome), and Lennox–Gastaut syndrome, fall within the spectrum of DEEs [12].

Early onset of seizures has been shown to be an important risk factor for DRE [13]. Early-onset epilepsy is often caused by genetic disorders and structural abnormalities, such as congenital brain malformations and perinatal brain injuries [14]. Identifying the etiology of epilepsy is important for understanding the underlying mechanism and enables the use of alternative treatments in drug-resistant cases [15].

This study is a continuation of our earlier nationwide, population-based investigation of the incidence and etiology of DRE in children and adolescents aged ≤18 years [16]. In that study, DRE was identified in 10% of patients, which is lower than commonly reported in the literature and likely reflects the comprehensive nationwide management of pediatric epilepsy within Estonia’s centralized tertiary care system [16]. Building on these findings, the present study aims to further characterize epilepsy syndromes, comorbidities, incidence, and the etiology of early-onset DRE, defined as epilepsy with onset before two years of age.

## 2. Materials and Methods

### 2.1. Study Cohort

The cohort comprised all children permanently residing in Estonia who were diagnosed with epilepsy before the age of two years between 1 January 2013 and 31 December 2017 and who met the ILAE criteria for DRE. According to these criteria, DRE was defined as the failure of adequate trials of two tolerated and appropriately chosen ASM regimens, resulting in the initiation of a third [1]. In this study, these patients are referred to as early-onset DRE (epilepsy onset before two years of age).

### 2.2. Case Ascertainment

Estonia has a population of approximately 1.36 million and has and only two pediatric neurology departments—at the Children’s Clinic of Tartu University Hospital and Tallinn Children’s Hospital, both of which are tertiary care centers. Most pediatric neurologists in Estonia work at these hospitals, and according to national consensus guidelines, epilepsy in children is diagnosed exclusively by pediatric neurologists at these centers. Therefore, this study includes all newly diagnosed pediatric DRE cases meeting the ILAE criteria nationwide [16].

Given the centralized structure of pediatric epilepsy care in Estonia, it is highly unlikely that children with DRE were managed outside these two tertiary centers. However, although the majority of pediatric epilepsy cases in Estonia are managed in these two tertiary centers, some children with drug-responsive epilepsy may not have been captured in this cohort; this is expected to represent only a small proportion given the centralized referral system for pediatric epilepsy care in Estonia.

Patients were initially identified through hospital-based electronic registries from both tertiary centers. As previously described in our earlier publication, we retrieved comprehensive datasets of all individuals aged ≤18 years who received a first-time diagnosis of epilepsy between 1 January 2013 and 31 December 2017. Case identification was performed using International Classification of Diseases 10th Revision (ICD-10) codes G40 and G41 from hospital electronic medical records [16].

All epilepsy diagnoses were subsequently confirmed by a pediatric neurologist in accordance with the ILAE operational definition of epilepsy, which includes at least two unprovoked (or reflex) seizures occurring more than 24 h apart, a single unprovoked seizure with an estimated ≥60% risk of recurrence, or a diagnosis of an accepted epilepsy syndrome [17].

To ensure data validity, all medical records were manually reviewed in detail from the time of initial diagnosis through 1 September 2020. This review confirmed that the recorded year of epilepsy onset fell within the predefined study period and that the diagnosis of epilepsy was definite rather than suspected or later disproved. Among confirmed incident epilepsy cases, those who subsequently developed DRE were identified [16]. DRE was defined as failure to achieve sustained seizure freedom despite adequate trials of two appropriately selected and tolerated antiseizure medication (ASM) regimens, after which a third ASM was initiated due to lack of efficacy [1].

ASM treatment decisions were guided by national and international clinical recommendations, while allowing for individual clinical judgment based on patient-specific factors. Variability in clinical practice may have influenced the timing of introduction of a third ASM; however, the definition of DRE was applied consistently according to ILAE criteria.

For the present analysis, we focused on the subgroup of patients with early-onset DRE. Patients were included in the study if they had epilepsy onset before two years of age, met the ILAE criteria for DRE, and had permanent residence in Estonia, ensuring continuous access to healthcare services regardless of ethnic background.

### 2.3. Clinical Information and Results of Etiological Investigations

#### 2.3.1. Data Collection

Clinical data were collected through a detailed review of digital medical records, including medical history, clinical examination findings, seizure characteristics, age at epilepsy diagnosis, epilepsy syndrome classification, and treatment history.

#### 2.3.2. Cognitive and Neurodiagnostic Assessments

Results from repeated assessments—including cognitive evaluations, electroencephalography (EEG), and magnetic resonance imaging (MRI)—were reviewed and recorded.

#### 2.3.3. Genetic Testing

Genetic testing was performed based on clinical indications, guided by factors such as seizure type, age at onset, family history, and suspected etiology. Testing included chromosomal microarray analysis (CMA) and next-generation sequencing (NGS) approaches—including targeted gene panels, whole-exome sequencing (WES), and whole-genome sequencing (WGS)—as well as, in selected cases, single-gene sequencing or karyotyping. All research-derived results were validated in accredited medical laboratories [16].

Most testing was performed at the Genetics and Personalized Medicine Clinic, Tartu University Hospital, Estonia and was covered by Estonian Health Insurance Fund. A few single-gene and targeted gene panel tests were initially performed in commercial laboratories abroad before such testing became available in Estonia, and WGS was conducted in research setting at the Broad Institute of MIT and Harvard, Cambridge, MA, USA. Financial considerations did not influence test selection, as decisions were made solely based on clinical necessity [16].

While genetic testing is especially informative for Dravet syndrome, it can also be applied in IESS (including West syndrome), and Lennox–Gastaut syndrome, though their heterogeneous etiologies typically result in lower diagnostic yield.

Genetic testing approaches differed between patients and also evolved over the study period, with a shift from more targeted testing toward NGS methods over time. This variation may have influenced the overall diagnostic yield.

#### 2.3.4. Metabolic Testing

Selected metabolic investigations were performed where clinically indicated. These included, to varying extents across individual patients: analyses of amino acids and organic acids in serum and urine; purines, pyrimidines, as well as creatine and guanidinoacetate; glycosaminoglycans, monosaccharides, oligosaccharides, and sialic acid; urinary metabolic screening; transferrin analysis in serum for congenital disorders of glycosylation; and comprehensive analysis of folate derivatives, biopterins, neopterin, and neurotransmitters in cerebrospinal fluid.

#### 2.3.5. Epilepsy Syndrome and Etiology Classification

Epilepsy syndromes were classified according to the ILAE 2022 classification of epilepsy syndromes with onset in neonates and infants [2]. The screened DEEs included early-infantile DEE (formerly Ohtahara syndrome), epilepsy of infancy with migrating focal seizures, IESS (including West syndrome), and Dravet syndrome. Etiology-specific syndromes were defined according to the ILAE 2022 classification and included, for example, potassium-voltage-gated channel subfamily Q member 2-related-DEE (*KCNQ2*-DEE), cyclin-dependent kinase-like 5-related-DEE (*CDKL5*-DEE), *PCDH19* clustering epilepsy, glucose transporter type 1 deficiency syndrome, Sturge–Weber syndrome, gelastic seizures associated with hypothalamic hamartoma, and pyridoxine-dependent-DEE [2]. If a patient’s presentation did not correspond to any listed DEE syndrome, it was designated as other DEE.

Epilepsy etiology was classified according to the ILAE 2017 scheme into six principal groups: structural, genetic, infectious, metabolic, immune, and unknown; in some patients, a combined etiology was assigned when more than one contributing cause (e.g., structural and genetic) was identified [11].

All available clinical, neuroimaging, neurophysiological, genetic, and metabolic data were integrated to determine the final epilepsy syndrome and etiological classification for each patient.

### 2.4. Statistical Methods

DRE incidence rates were determined by the number of newly diagnosed cases per 100,000 person-years. The study population included all children under two years of age at the beginning of each year, based on data from Statistics Estonia (www.stat.ee, accessed on 15 January 2026).

Between 2013 and 2017, the average annual population of children under two years of age in Estonia was 27,944, with minimal variation over the study period (range: 27,242–28,746). Over the five-year study period, this corresponded to a total of 139,718 person-years at risk.

Age- and sex-specific incidence rates were calculated using the same method. Ninety-five percent confidence intervals (95% CI) for incidence rates were estimated assuming a Poisson distribution.

## 3. Results

### 3.1. Patient Population and Birth Characteristics

In our previous nationwide study, 110 patients under 19 years of age were diagnosed with DRE in Estonia between 2013 and 2017, and the overall incidence and etiological distribution were described [16]. The present study continues this work, focusing on the subgroup with early-onset DRE. Of the 110 patients in the original cohort, 37 (34%) met this criterion and were included in the current analysis.

Within this subgroup, 54% (*n* = 20) were female and 46% (*n* = 17) were male. Epilepsy was diagnosed before the age of one year in 68% of cases (*n* = 25), and between one and two years of age in 32% (*n* = 12). The mean age at diagnosis was nine months, and the median age was seven months (range: one to 23 months).

Most patients were born at term (81%, *n* = 30). Six patients (16%) were born preterm, including one late preterm (34–<37 weeks), two moderate preterm (32–<34 weeks), two very preterm (28–<32 weeks), and one extremely preterm (<28 weeks), according to the World Health Organization classification of preterm birth [18]. One patient (3%) was born post-term (≥42 weeks of gestation).

Severe perinatal asphyxia was documented in two patients (5%); Apgar scores at 1, 5, and 10 min were 1, 3, and 5, respectively. One term-born patient developed hypoxic–ischemic perinatal injury evident on MRI. The other patient, born very preterm (at 29 weeks’ gestation), had MRI findings of white matter injury and intraventricular hemorrhage, consistent with complications of prematurity rather than hypoxic–ischemic encephalopathy. However, a contribution from perinatal asphyxia cannot be excluded. The clinical, demographic, and birth characteristics of the early-onset DRE cohort are summarized in Table 1.

### 3.2. Incidence

The overall incidence of DRE among children whose epilepsy was diagnosed before two years of age was 26.5 per 100,000 person-years (95% CI 18.7–36.5). The incidence was 23.7 per 100,000 person-years in males (95% CI 13.8–37.9) and 29.4 per 100,000 person-years in females (95% CI 18.0–45.5).

The highest incidence was observed during the first year of life, reaching 36.1 per 100,000 person-years, and declined after the first birthday to 17.1 per 100,000 person-years among children aged 12 to 23 months. Age-and sex-specific incidence rates of early-onset DRE are presented in Table 2.

### 3.3. Treatment and Drug Resistance

Prior to the development of drug resistance (during the first two treatment regimens), patients were treated with various ASMs, administered either as monotherapy or combination therapy, including valproic acid, carbamazepine, oxcarbazepine, phenobarbital, levetiracetam, vigabatrin, lamotrigine, clobazam, nitrazepam, and glucocorticosteroids. From the third treatment regimen onwards, additional treatment modalities were introduced, including stiripentol, sulthiame, topiramate, phenytoin, clonazepam, everolimus, a ketogenic diet, vagus nerve stimulation (VNS), and surgical treatment.

Valproic acid was the most frequently used initial ASM following seizure onset, administered in 62% of patients (*n* = 23). Phenobarbital and levetiracetam were each used as initial therapy in 11% of patients (*n* = 4), followed by vigabatrin in 8% (*n* = 3), oxcarbazepine in 5% (*n* = 2), and glucocorticosteroids in 3% (*n* = 1).

Glucocorticosteroid pulse therapy was administered to 21 of 37 patients (57%) with DRE during their disease, primarily for indications such as infantile spasms, electrical status epilepticus in sleep (ESES), or frequent serial seizures.

Three patients (8% of the total cohort) with focal DRE underwent surgical treatment: one with hemimegalencephaly, one with focal cortical dysplasia, and one with a brain tumor. Additionally, two patients (5%) received VNS implants. Five additional children with DEEs (14% of the total cohort) had tried a ketogenic diet as an alternative treatment to control seizures.

Drug resistance developed within six months of epilepsy diagnosis in 43% of patients, and within one year in nearly two-thirds (65%) of cases (Table 3).

The mean time to development of drug resistance—defined as the time at which the third ASM regimen was initiated—was 11 months, with a median of eight months. The mean age at which epilepsy was classified as drug-resistant was 20 months, while the median age was 17 months.

### 3.4. Epilepsy Syndromes

Among the 37 patients with early-onset DRE, epilepsy syndromes were identified in 30 patients (81%) according to the ILAE 2022 classification. Of these, 28 patients (76%) had DEE, while two patients (5%) had an etiology-specific epilepsy syndrome without DEE (*PCDH19* clustering epilepsy). The remaining seven patients (19%) presented with focal DRE without a specific epilepsy syndrome.

Patients with DEE were further categorized by subtype, with IESS (including West syndrome) being the most common, occurring in almost half of the DEE group (13 of 28 patients, 46%) and in about a third of the total early-onset DRE cohort (35%). 11 of the 28 patients with DEE (39% of DEE; 30% of DRE) could not be assigned to any specific syndrome defined in the ILAE 2022 classification and were therefore categorized as having other DEEs. Dravet syndrome was diagnosed in two patients. Etiology-specific DEEs were identified in two additional patients (one with *CDKL5*-DEE and the other with *KCNQ2*-DEE). Table 4 presents the distribution of DEE subtypes and their proportions among all DEE and DRE patients.

### 3.5. Neuroimaging

Brain MRI was performed in all DRE patients (*n* = 37) as part of the diagnostic evaluation. A 3.0 Tesla MRI was conducted in 27% of patients (*n* = 10), while the remaining 73% of patients (*n* = 27) underwent 1.5 Tesla MRI, depending on availability.

Overall, 46% of patients (*n* = 17) had either negative or nonspecific MRI findings. Of these, 32% (*n* = 12) had negative MRI findings, while nonspecific neuroimaging abnormalities were observed in 14% (*n* = 5), including benign enlargement of the subarachnoid space in infancy, hippocampal malrotation, and small benign cysts. Progressive brain atrophy without other structural pathology was observed in 5% of patients (*n* = 2) on follow-up MRIs. Both patients had a genetic etiology for their epilepsy: one had a de novo pathogenic variant in *CDKL5* and the other in *DNM1*, which may have contributed to the development of atrophy [16].

In 49% of patients (*n* = 18), structural abnormalities of etiological relevance were identified. In the early-onset DRE cohort, congenital brain malformations were the most frequent findings, observed in 22% (*n* = 8) of patients. Additionally, two patients (5%) had inflicted traumatic brain injury, and two others (5%) exhibited preterm-related white matter injury with intraventricular hemorrhage. Single cases were identified with perinatal hemorrhagic stroke, hypoxic–ischemic perinatal injury, brain tumor, tuberous sclerosis complex, porencephaly followed Herpes Simplex Virus Type 2 (HSV-2)-encephalitis, and mesial temporal sclerosis.

Based on the epilepsy type or syndrome, MRI revealed etiologically relevant structural pathology in four of seven patients (57%) with focal epilepsy and in 14 of 28 patients (50%) with DEE, whereas none of the patients with *PCDH19* clustering epilepsy showed MRI abnormalities. In focal epilepsy, congenital brain malformations were identified in two cases (focal cortical dysplasia and hemimegalencephaly), while single cases included preterm-related white matter injury with intraventricular hemorrhage and a brain tumor. In DEE, congenital brain malformations were the most common finding, followed by inflicted traumatic brain injury and isolated cases of other structural etiologies.

The distribution of MRI findings across all DRE patients, categorized by epilepsy type or syndrome, is summarized in Table 5.

### 3.6. Genetic Testing

Genetic testing included targeted gene panels, WES, and/or WGS, as well as CMA and, in selected cases, single gene testing and karyotyping. One or more genetic tests were performed in 34 of 37 patients (92%) with early-onset DRE.

CMA was conducted in 26 patients (70% of the total cohort), and targeted gene panels were used in an equal proportion (*n* = 26, 70%). WES and/or WGS were performed in 13 patients (35%), karyotyping in five patients (14%), and single-gene sequencing in one patient (3%). The extent of genetic testing performed for each patient with DRE and categorized by epilepsy type or syndrome is detailed in Table 6. The managing child neurologist and/or clinical geneticist selected the testing approach.

The most frequently performed genetic tests and their combinations are shown below. CMA and targeted gene panels were conducted together in nine patients (24% of the total cohort). Gene panels alone were performed in seven patients (19%), whereas six patients (16%) underwent CMA, gene panel, and WES/WGS. Overall, 24 of 37 patients (65% of the total cohort) had more than one genetic test, highlighting the frequent use of combined testing strategies.

In three patients (8%), genetic testing was not performed, as the etiology of their epilepsy had already been established. All three patients had structural brain abnormalities (brain tumor, traumatic brain injury, or congenital brain malformation). Therefore, genetic testing was not considered clinically indicated and was not part of routine diagnostic management in these cases.

Of the 34 patients with DRE who underwent genetic testing, 12 (35%) showed normal results, which included patients with no variants detected and those with only benign or likely benign variants. Among the total cohort of 37 patients, pathogenic genetic findings were identified in 14 (38%). These comprised 13 pathogenic sequence variants, classified according to the American College of Medical Genetics and Genomics (ACMG) criteria, accounting for 35% of the total cohort, and one chromosomal aberration (ring chromosome 14) identified in one patient (3%). Variants of uncertain significance (VUS) were detected in four patients (11% of the total cohort). Reanalysis of the genetic data identified novel disease genes in four patients (11%), one of which, *RNU2-2*, was later reclassified as pathogenic. With the inclusion of the pathogenic *RNU2-2* variant, the proportion of patients with pathogenic genetic findings increases from 38% to 41%.

It should be noted that the genetic results for these patients have been previously reported as part of a larger DRE cohort including patients up to 19 years of age [16]. In the present study, we focus on a subcohort of early-onset DRE patients. The phenotype and genotype of the patient with a pathogenic *CDKL5* variant have also been reported previously [19]. Table 7 provides information on sequence variants only, including pathogenic variants, VUS, and novel disease genes, and therefore does not include the chromosomal aberration (ring chromosome 14). Supplementary Table S1 provides a comprehensive list of all sequence variants, including both nucleotide and protein changes, as well as the chromosomal aberration.

#### Genetic Findings by Epilepsy Type or Syndrome

One or more genetic analyses were performed in all patients with *PCDH19* clustering epilepsy, in 27 of 28 patients (96%) with DEEs, and in five of seven patients (71%) with focal epilepsy. All three patients who did not undergo genetic testing had structural etiology for their DRE (Table 6).

All patients with *PCDH19* clustering epilepsy (*n* = 2) carried a pathogenic variant in the *PCDH19* gene. In contrast, among seven patients with focal epilepsy, only one (14%) harbored a pathogenic variant, which was located in the *PRRT2* gene. Variants in two potential novel disease genes, *DSCAM* and *LMTK3*, were also identified in one patient (14%) with focal epilepsy; however, their pathogenic role has not yet been confirmed.

Pathogenic genetic findings were identified in 11 of 28 patients (39%) with DEEs. Ten patients had pathogenic variants in the following genes: *CDKL5*, *COL4A1*, *DNM1*, *GABRG2*, *IRF2BPL*, *KCNQ2*, *KMT2D*, *SCN1A*, *SYNGAP1*, and *TSC2.* Additionally, one patient carried a de novo ring chromosome 14. Variants in novel disease genes were identified in three patients with DEEs (11%). A *SIRT6* variant was detected in two siblings with DRE from a set of triplets; however, its pathogenicity has not yet been confirmed, as functional studies are ongoing. In another patient, variants in *ACSL5* and *RNU2-2* genes were identified, with *ACSL5* classified as VUS and *RNU2-2* later reclassified as pathogenic, raising the proportion of patients with DEEs with pathogenic genetic findings from 39% to 43%.

### 3.7. Metabolic Testing

Metabolic investigations were performed in all eight patients with DRE of unknown etiology, but no significant abnormalities were found.

### 3.8. Comorbidities

A significant proportion of patients with DRE (*n* = 32, 86%) had GDD or ID. Additionally, four patients (11%) presented with isolated speech and language delay without GDD or ID. Age-appropriate speech, language, cognitive, and motor development was observed in only one patient (3%). Among those with ID, two children (5% of the total cohort) were also diagnosed with attention-deficit/hyperactivity disorder (ADHD), and one child (3%) had co-occurring ADHD and autism spectrum disorder.

Of the 37 patients with DRE, 17 (46%) exhibited motor impairments. Among these, tetraparesis was present in 13 (76%), spastic hemiparesis in two (12%), and spastic diplegia in two (12%). Six patients (16%) with DRE, whose epilepsy onset occurred before the age of two years, died between the ages of 10 months and eight years. All of these patients had severe GDD, tetraparesis, and an underlying genetic, structural, or combined etiology.

### 3.9. Etiology

The etiology of early-onset DRE was identified in 29 of 37 children (78%). In the remaining eight patients (22%), the etiology could not be determined. This subgroup included three patients (8% of the total cohort) without GDD or ID and five patients (14%) with GDD or ID. In the latter group, the underlying cause remained unresolved; however, two patients carried VUS, and one patient had a novel disease gene.

**Figure 1:**
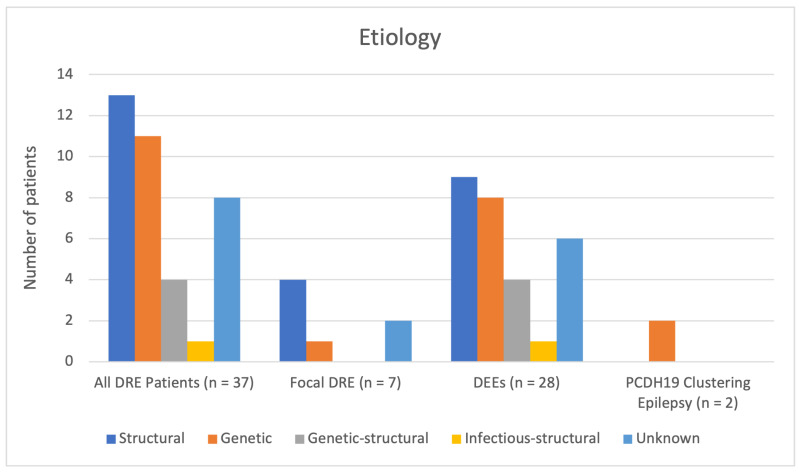
Etiological distribution of all early-onset DRE patients and categorized by epilepsy type or syndrome.

The most common etiologies in the total DRE cohort were structural (*n* = 13, 35%) and genetic (*n* = 11, 30%). A combined genetic–structural etiology was identified in four patients (11%). One patient had an infectious–structural etiology due to HSV-2 encephalitis resulting in porencephaly. Figure 1 presents the etiological distribution of all early-onset DRE patients, stratified by epilepsy type or syndrome.

Table 8 presents the exact structural, genetic, combined genetic–structural, and infectious–structural causes in all early-onset DRE patients, as well as by epilepsy type or syndrome. Unknown etiologies are stratified by the presence or absence of GDD/ID. A detailed analysis of etiologies by epilepsy type or syndrome is provided in a subsequent section.

#### Etiology by Epilepsy Type or Syndrome

As shown in Figure 1 and Table 8, an underlying etiology was identified in five of seven patients (71%) with focal DRE and in 22 of 28 patients (79%) with DEEs. In both patients with *PCDH19* clustering epilepsy, a genetic etiology was confirmed based on pathogenic de novo variants in the *PCDH19* gene; neither had GDD or ID.

Among patients with focal DRE, four of seven (57%) had a structural etiology and one (14%) had a genetic etiology, involving a pathogenic variant in the *PRRT2* gene. Structural etiologies included congenital brain malformations in two patients (hemimegalencephaly in one and focal cortical dysplasia in the other). One very preterm infant had white matter injury with intraventricular hemorrhage, and an additional patient presented with a brain tumor. In the remaining two patients with focal DRE, the etiology remained unidentified; however, neither had GDD nor ID.

Among patients with DEEs, the most common etiologies were structural (32%, *n* = 9) and genetic (29%, *n* = 8). Within the structural etiology group, five patients had congenital brain malformations, two had inflicted traumatic brain injury, one had preterm-related white matter injury with intraventricular hemorrhage, and one had hypoxic–ischemic perinatal injury. In the genetic etiology group, seven patients with DEE carried pathogenic variants in the following genes: *CDKL5* [19], *DNM1*, *GABRG2*, *IRF2BPL*, *KCNQ2*, *SCN1A*, *SYNGAP1*; one patient had a chromosomal aberration (ring chromosome 14).

A combined genetic-structural etiology was identified in four patients with DEE (14%). These included a pathogenic maternal variant in the *COL4A1* gene resulting in perinatal hemorrhagic stroke, a pathogenic de novo variant in the *KMT2D* gene associated with a congenital brain malformation, and a pathogenic de novo variant in the *TSC2* gene causing tuberous sclerosis complex. In one patient, a pathogenic variant in the novel disease gene *RNU2-2* and mesial temporal sclerosis on MRI were identified. A combined infectious-structural etiology (4%) was identified in one patient with HSV-2 encephalitis resulting in porencephaly.

In six of 28 patients with DEE (21%), the etiology remained unidentified, including one patient without GDD or ID and five patients with GDD or ID. One patient with IESS (a DEE subtype) became seizure-free after treatment with three ASMs and did not develop GDD or ID. In contrast, the remaining five patients with DEE of unknown etiology had GDD or ID, suggesting an as-yet unidentified underlying cause.

**Figure 2:**
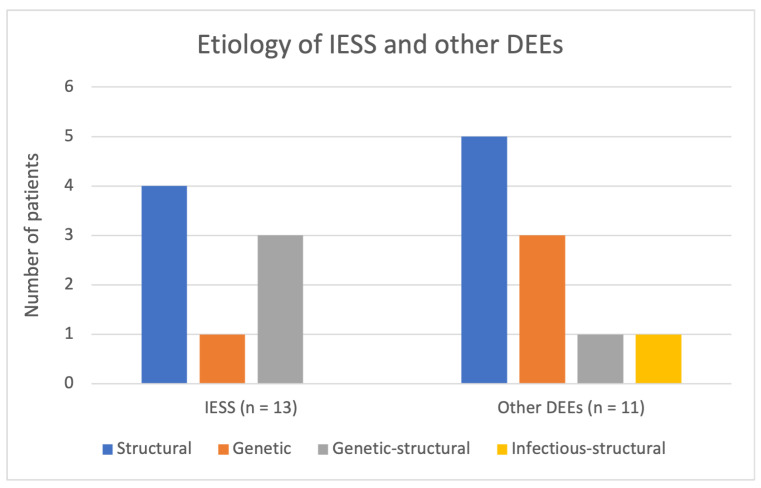
Etiological distribution of IESS and other DEEs.

Etiology was further analyzed within DEE subgroups, including IESS, other DEEs, and etiology-specific epilepsy syndromes. Four patients had syndromes with a well-established genetic basis according to the ILAE classification. Pathogenic variants in *SCN1A*, a common cause, and *GABRG2*, a rare but recognized cause, were identified in two patients diagnosed with Dravet syndrome. In addition, one patient had *CDKL5*-DEE and one had *KCNQ2*-DEE, each associated with a pathogenic variant in the corresponding gene. Figure 2 summarizes the etiologic distribution for IESS and other DEEs.

As presented in Figure 2, among patients with IESS, structural etiologies were most common, identified in four of 13 patients (31%), followed by genetic–structural causes in three patients (23%), and a purely genetic cause in one patient (8%), resulting in a known etiology in 62% of cases. Among IESS patients with structural etiologies, two presented with inflicted traumatic brain injury, one with a congenital brain malformation, and another with preterm-related white matter injury with intraventricular hemorrhage. In IESS patients with combined genetic-structural etiologies, the findings overlapped with those described above and included *COL4A1*-associated perinatal hemorrhagic stroke, *KMT2D*-associated congenital brain malformation, and *TSC2*-associated tuberous sclerosis complex. The single IESS patient with a purely genetic etiology carried a pathogenic de novo variant in *IRF2BPL*. Among patients with other DEEs, structural etiologies were the most common (45%), followed by genetic causes (27%), combined genetic-structural (9%), and infectious–structural etiologies (9%).

Overall, early-onset drug-resistant epilepsy in this nationwide cohort was predominantly characterized by developmental and epileptic encephalopathies, with a heterogeneous etiological spectrum dominated by structural and genetic causes. It was associated with rapid progression to drug resistance and a high burden of neurodevelopmental comorbidities.

## 4. Discussion

This study represents a continuation of our nationwide, population-based investigation of DRE in patients with epilepsy onset before 19 years of age between 2013 and 2017. The present analysis focuses specifically on early-onset DRE. We further examined the etiology of DRE across different epilepsy types and syndromes. This age group is of particular importance, as epilepsy incidence is highest during the first year of life and early-onset epilepsy carries a substantially increased risk of drug resistance.

We found that the incidence of DRE with epilepsy onset before two years of age was 26.5 per 100,000 person-years. The rate was approximately twice as high among children diagnosed before one year of age (36.1 per 100,000 person-years) compared to those diagnosed between 12 and 23 months (17.1 per 100,000 person-years). Overall, the incidence in children under two years exceeded that observed in older age groups in our previous study (17.7 per 100,000 person-years for children up to five years and 8.5 per 100,000 person years for those up to 19 years) [16]. Notably, the rate among children aged 12 to 23 months was similar to that reported previously for the broader age group up to five years (17.1 vs. 17.7 per 100,000 person-years, respectively) [16]. This likely reflects the well-established pattern that both epilepsy incidence and the risk of developing DRE peak during infancy and decline thereafter [5,13,14,20,21].

Although several epidemiological studies have reported the incidence of epilepsy in children under two years of age, few have provided specific data on DRE in this age group. A Korean study similarly demonstrated the highest DRE incidence during infancy (21.15 per 100,000 person-years in children under one year), with declining rates at one and two years of age (12.31 and 7.10 per 100,000 person-years, respectively) [22]. In contrast, Symonds et al. reported a cumulative incidence of 82.0 per 100,000 live births for DRE with epilepsy onset before three years of age, although person-year-based estimates were not provided [14]. Although the studies differ in design and methods of calculating incidence, they consistently show that the burden of DRE is highest in infancy. Overall, directly comparable population-based incidence data for DRE in very young children remain limited.

Drug resistance developed rapidly in our cohort: 43% of patients met criteria within six months and 65% within one year of diagnosis. This rapid progression is consistent with previous reports indicating that DRE can often be recognized early in the course of treatment, supporting the concept that drug resistance is frequently present from disease onset [23,24,25].

Epilepsy syndromes were identified in 81% of patients according to the ILAE 2022 classification. Of these, 76% were classified as DEEs and 5% as etiology-specific epilepsy syndromes without DEE (*PCDH19* clustering epilepsy). IESS (including West syndrome) was the most common DEE subtype, accounting for nearly half of the DEE cases and approximately one-third of the entire early-onset DRE cohort. A Swedish study of children with epilepsy onset before two years—without restriction to drug-resistant cases—identified epilepsy syndromes in 54% of patients [21]. Vikin et al. in a cohort not restricted to DRE, reported that DEEs were most prevalent in early-onset epilepsies, occurring in 72% of cases with onset before one year and 47% of cases with onset between one and two years [5]. In the same study, among epilepsies beginning in the first year of life, 44% were classified as specific ILAE-defined DEE syndromes, most commonly IESS [5]. Although cohort characteristics differ, all studies point to a high prevalence of DEEs in early-onset epilepsy, particularly IESS, highlighting the importance of early recognition and targeted evaluation.

Structural abnormalities were prominent in our cohort. Structurally relevant abnormalities were identified in 49% of early-onset DRE patients, including congenital malformations, perinatal and traumatic injuries, tuberous sclerosis, and mesial temporal sclerosis. Similarly, Yildiz et al. reported structural etiologies as the most frequent cause (33.6%) among children with-early onset DRE, with perinatal brain injury and malformations of cortical development predominating [26]. These findings highlight continued importance of neuroimaging in the evaluation of early-onset DRE.

Pathogenic genetic findings were identified in 41% of our DRE cohort, with pathogenic sequence variants in 38% (including the novel disease gene *RNU2-2*) and a chromosomal aberration—a ring chromosome 14—in 3%. This proportion exceeds the 15% reported by Yildiz et al. [26] and likely reflects broader implementation of next-generation sequencing in our cohort. Notably, genetic defects have been shown to significantly increase the risk of DRE in young children with epilepsy and neurodevelopmental disability, highlighting the critical role of genetics in early-onset DRE [27].

These findings highlight the substantial contribution of genetic factors to early-onset DRE and support the relevance of underlying molecular mechanisms.

Although the identified pathogenic variants are genetically heterogeneous, they align with a limited number of shared biological mechanisms, including altered neuronal excitability, synaptic dysfunction, and disrupted neurodevelopment. These mechanisms involve distinct functional gene groups, comprising ion channel activity, synaptic transmission, and neurodevelopmental processes.

*SCN1A*, *KCNQ2*, and *GABRG2* variants affect ion channel function and inhibitory signaling, resulting in increased neuronal excitability through disruption of excitation–inhibition balance [28]. *DNM1* encodes a neuronal GTPase essential for synaptic vesicle recycling, and pathogenic variants disrupt vesicle trafficking, resulting in synaptic dysfunction and neuronal hyperexcitability [29]. Similarly, *SYNGAP1* encodes a postsynaptic regulator of excitatory signaling, and its haploinsufficiency disrupts synaptic plasticity and network stability, contributing to epilepsy [30].

*CDKL5*, *TSC2*, *KMT2D*, and *IRF2BPL* are involved in transcriptional regulation, intracellular signaling, and neuronal development. *CDKL5* is essential for neuronal network development, while *TSC2* regulates the mTOR pathway, a key regulator of cell growth and metabolism; its dysfunction is associated with abnormal neuroplasticity, tumor formation, and increased neuronal excitability [31,32]. *KMT2D* contributes to chromatin remodeling and gene expression, while *IRF2BPL* is implicated in neuronal function and maintenance, with disruption of these genes leading to neurodevelopmental dysfunction and neurodegeneration [33,34].

Collectively, these findings suggest that despite genetic heterogeneity, the underlying mechanisms converge on impaired neuronal excitability, synaptic dysfunction, and disrupted neurodevelopment, which likely contribute to the refractory nature of epilepsies in these patients.

Overall, an underlying etiology was identified in 78% of our early-onset DRE cohort. This is higher than the 64.3% reported by Yildiz et al. and slightly lower than the 82% reported by Symonds et al.; in both studies, the analyses specifically considered children who developed DRE, with epilepsy onset before two and three years, respectively [14,26]. Differences in diagnostic strategies, availability of genetic testing, and age cut-offs may explain these variations.

In our total DRE cohort, the most common etiologies were structural (35%) and genetic (30%), with combined genetic–structural causes identified in 11%. Stödberg et al. reported structural etiologies in 34% of cases and genetic etiologies in 20% of children with epilepsy onset in the first two years, although their study was not restricted to DRE [21]. Yildiz et al. found structural etiology (33.6%) and genetic (15%) causes being the most common, followed by metabolic (10.7%) and infectious (5%) in children with epilepsy onset before two years who developed DRE [26]. A Scottish population-based study of children with epilepsy onset before three years found genetic etiologies in 52.5% and structural causes in 26.6% of cases among those who developed DRE [14], illustrating variability across populations.

Overall, these findings highlight both the substantial contribution of structural and genetic factors to early-onset DRE and the considerable variability in reported frequencies across different populations and study designs, emphasizing the need for comprehensive, population-specific etiological assessment.

Among the subgroup of patients with DEEs within our early-onset DRE cohort, an underlying etiology was identified in 79%, exceeding the approximately 50% diagnostic yield often reported in the literature [35]. This high detection rate likely reflects the systematic and comprehensive etiological evaluation performed in our study.

Although DEEs are predominantly considered genetic disorders—with more than 800 monogenic epilepsy genes currently implicated [36]—structural, metabolic, and other acquired etiologies also contribute substantially to the epileptic and developmental phenotype [35]. In our DEE subgroup, structural causes were the most frequent (32%), including congenital brain malformations and acquired brain injuries associated with severe epileptic phenotypes and poor neurodevelopmental outcomes. Genetic etiologies accounted for 29% of cases and comprised several well-established DEE-associated genes, as well as a chromosomal aberration, consistent with previous reports [36,37]. Combined etiologies, most often genetic-structural (14%) in our DEE subcohort, further illustrate the heterogeneity and multifactorial nature of DEEs.

Collectively, these findings emphasize the importance of early and comprehensive diagnostic evaluation, which is critical for improving prognosis and guiding the development of targeted therapies.

Neurodevelopmental comorbidity was substantial. GDD or ID was present in 86% of our patients with early-onset DRE, comparable to the 83% reported by Symonds et al. in children with seizure onset before three years who developed DRE [14]. This indicates that very early-onset DRE represents a severe neurodevelopmental disorder rather than an isolated seizure condition.

This study is limited by a restricted cohort size, which mirrors the small size of the underlying population. This may have reduced the statistical power of subgroup analyses, and therefore some differences between smaller etiological groups should be interpreted with caution. In addition, the generalizability of the findings to other populations may be limited.

Despite this, our study included all pediatric neurology centers in Estonia that manage children with epilepsy, both of which also serve as tertiary care hospitals. Some newly diagnosed patients with non–drug-resistant epilepsy may not have been captured in our cohort. Nevertheless, given that Estonia’s healthcare system allows unrestricted patient movement between centers and that families of children with refractory epilepsy typically seek specialist care, it is highly unlikely that a significant number of children with DRE would remain undiagnosed or untreated in specialized epilepsy centers.

A limitation of the study is that newer antiseizure medications, for example lacosamide, brivaracetam, and cannabidiol, were not available in our treatment protocol during the study period.

Another potential limitation concerns the estimation of genetic etiology. Four patients initially carried variants in novel disease genes, one of which (*RNU2-2*) has since been reclassified as pathogenic. This reclassification is supported by recent publications [38,39], as well as by its inclusion in the Online Mendelian Inheritance in Man (OMIM) database, and two entries in the ClinVar database. At the time of preparing our previous manuscript, this gene was still considered a potential novel disease gene. This highlights the evolving nature of genetic knowledge, as findings initially considered uncertain may later be established as pathogenic. As additional functional and clinical data become available, the proportion of patients with a confirmed genetic etiology may increase.

Furthermore, not all patients underwent identical genetic and metabolic analyses, since diagnostic evaluations and treatment decisions were made by each neurologist or geneticist according to their best clinical judgment in routine practice. On the other hand, genetic testing forms an essential part of routine care for children with epilepsy—particularly for those with DRE and DEE—and is widely available and reimbursed within the Estonian healthcare system. One or more genetic tests were performed in almost all (92%) patients with DRE, with only three patients with known structural etiologies not undergoing genetic testing. This supports the reliability of our genetic findings despite these limitations.

A strength of the study is that all DRE patients underwent MRI. However, 3.0 Tesla MRI was performed in only 27% of patients due to availability, while the remaining patients had 1.5 Tesla MRI. Increasing the proportion of patients scanned with 3.0 Tesla MRI might improve the detection of structural etiologies.

Overall, despite the small population size, Estonia’s nationwide, well-digitalized medical system and inclusion of all pediatric epilepsy centers allow for reliable population-based epidemiological research. These structural advantages reduce the impact of the study’s limitations.

## 5. Conclusions

In conclusion, early-onset DRE in Estonia is characterized by a particularly high incidence during the first year of life, with rapid progression to drug resistance, predominance of DEEs, and substantial neurodevelopmental comorbidity. The high etiological yield—particularly for structural and genetic causes—emphasizes the importance of early, comprehensive diagnostic evaluation in understanding early-onset DRE. Few population-based studies have specifically examined early-onset DRE; most prior research has focused on early-onset epilepsy in general or on DEEs alone in this age group. Therefore, our findings provide valuable data on this underexplored population. However, the relatively small sample size should be considered when interpreting the generalizability of the results.

## Figures and Tables

**Table 1:** Clinical, demographic, and birth characteristics of the early-onset DRE cohort.

Characteristic	Value
Total cohort (*n*)	37
Female, *n* (%)	20 (54%)
Male, *n* (%)	17 (46%)
Age at epilepsy diagnosis <1 year	25 (68%)
Age at epilepsy diagnosis 1–2 years	12 (32%)
Mean age at epilepsy diagnosis	9 months
Median age at epilepsy diagnosis	7 months (range 1–23 months)
Born at term	30 (81%)
Preterm birth	6 (16%)
Post-term birth	1 (3%)
Severe perinatal asphyxia	2 (5%)

**Table 2:** Incidence rates of early-onset DRE per 100,000 person-years, stratified by age and sex across pediatric age groups.

Age (Months)	Total			Males			Females		
	Children (*n*)	Cases (*n*)	Rate (95% CI)	Children (*n*)	Cases (*n*)	Rate (95% CI)	Children (*n*)	Cases (*n*)	Rate (95% CI)
<12	69,349	25	36.1 (23.3–53.2)	35,694	11	30.8 (15.4–55.1)	33,655	14	41.6 (22.7–69.8)
12–23	70,369	12	17.1 (8.8–29.8)	36,082	6	16.6 (6.1–36.2)	34,287	6	17.5 (6.4–38.1)
Total	139,718	37	26.5 (18.7–36.5)	71,776	17	23.7 (13.8–37.9)	67,942	20	29.4 (18.0–45.5)

Children (*n*) indicates the cumulative number of children in each age group over the five-year period. Rates are reported per 100,000 person-years.

**Table 3:** Time to drug resistance from epilepsy diagnosis.

Time to Drug Resistance	Patients, *n* (%)
≤6 months	16 (43%)
7–12 months	8 (22%)
13–18 months	6 (16%)
19–24 months	1 (3%)
>24 months	6 (16%)

**Table 4:** Distribution of DEE subtypes among early-onset DRE patients.

DEE Subtype	Patients, *n* (% of DEE/% of DRE) *
IESS (including West syndrome)	13 (46%/35%)
Dravet syndrome	2 (7%/5%)
*CDKL5*-DEE	1 (4%/3%)
*KCNQ2*-DEE	1 (4%/3%)
Other DEEs	11 (39%/30%)

* Percentages represent (1) the proportion of all DEE patients (*n* = 28), and (2) the proportion of all DRE patients (*n* = 37). No patients in this cohort were diagnosed with Rasmussen’s encephalitis.

**Table 5:** MRI findings in all DRE patients and categorized by epilepsy type or syndrome.

Structural Pathology	All DRE Patients (*n* = 37)	Focal DRE (*n* = 7)	DEEs (*n* = 28)	*PCDH19* Clustering Epilepsy (*n* = 2)
Congenital brain malformations	8 *	2 *	6	0
Inflicted traumatic brain injury	2	0	2	0
Preterm-related white matter injury with intraventricular hemorrhage	2	1	1	0
Perinatal hemorrhagic stroke	1	0	1	0
Hypoxic–ischemic perinatal injury	1	0	1	0
Brain tumor	1	1	0	0
Tuberous sclerosis	1	0	1	0
Porencephaly followed HSV-2 encephalitis	1	0	1	0
Mesial temporal sclerosis	1	0	1	0
Brain atrophy without other structural abnormality	2	0	2	0
Negative or nonspecific findings	17	3	12	2

* Including one case of focal cortical dysplasia.

**Table 6:** Extent of genetic testing in all DRE patients and categorized by epilepsy type or syndrome.

Genetic Assay	All DRE Patients (*n* = 37)	Focal DRE (*n* = 7)	DEEs (*n* = 28)	*PCDH19* Clustering Epilepsy (*n* = 2)
CMA only	2	0	2	0
CMA and gene panel	9	2	7	0
CMA and WES/WGS	4	0	4	0
CMA, gene panel and WES/WGS	6	1	5	0
CMA, gene panel and karyotype	2	1	1	0
CMA, single gene testing and karyotype	1	0	1	0
CMA, gene panel, WES/WGS and karyotype	2	0	2	0
Gene panels only	7	1	4	2
WES only	1	0	1	0
Genetic testing not performed	3	2	1	0

**Table 7:** ACMG-based classification of single-gene sequence variants.

Sequence Variants	Patients (*n*)	Genes
Pathogenic	13	Two cases: *PCDH19* One patient: *CDKL5* [19], *COL4A1*, *DNM1*, *GABRG2*, *IRF2BPL*, *KCNQ2*, *KMT2D*, *PRRT2*, *SCN1A*, *SYNGAP1*, *TSC2*
Novel disease genes	4	Two cases: *SIRT6* One patient: *ACSL5*/*RNU2-2*, *DSCAM*/*LMTK3*
VUS	4	One patient: *SCN2A*, *SLC9A6*, *SPTAN1*, *WNK3*

**Table 8:** Etiology of all DRE patients and categorized by epilepsy type or syndrome.

Etiology	All DRE Patients (*n* = 37)	Focal DRE (*n* = 7)	DEEs (*n* = 28)	*PCDH19* Clustering Epilepsy (*n* = 2)
Structural:	13	4	9	0
Congenital brain malformations	7	2	5	0
Inflicted traumatic brain injury	2	0	2	0
Preterm-related white matter injury and intraventricular hemorrhage	2	1	1	0
Hypoxic–ischemic perinatal injury	1	0	1	0
Brain tumor	1	1	0	0
Genetic:	11	1	8	2
Pathogenic sequence variants	10	1	7	2
Chromosomal aberration	1	0	1	0
Genetic-structural:	4	0	4	0
*COL4A1*, perinatal hemorrhagic stroke	1	0	1	0
*KMT2D*, congenital brain malformation	1	0	1	0
*RNU2-2*, mesial temporal sclerosis	1	0	1	0
*TSC2*, tuberous sclerosis complex	1	0	1	0
Infectious-structural:	1	0	1	0
HSV-2 encephalitis, porencephaly	1	0	1	0
Unknown:	8	2	6	0
Without ID/GDD	3	2	1	0
With ID/GDD	5	0	5	0

## Data Availability

The original contributions presented in this study are included in the article and Supplementary Material. Further inquiries can be directed to the corresponding author.

## Supplementary Materials

The following supporting information can be downloaded at: https://www.mdpi.com/article/10.3390/neurolint18050076/s1, Table S1: A comprehensive list of all sequence variants, and the chromosomal aberration.

## Abbreviations

The following abbreviations are used in this manuscript:

ACMG	American College of Medical Genetics and Genomics
ADHD	Attention-deficit/hyperactivity disorder
ASM	Antiseizure medication
*CDKL5*	Cyclin-dependent kinase-like 5
CIs	Confidence intervals
CMA	Chromosomal microarray analysis
DEE	Developmental and epileptic encephalopathy
DRE	Drug-resistant epilepsy
EEG	Electroencephalography
ESES	Electrical status epilepticus in sleep
GDD	Global developmental delay
HSV2	Herpes Simplex Virus Type 2
ICD-10	International Classification of Diseases 10th Revision
ID	Intellectual disability
IESS	Infantile epileptic spasms syndrome
ILAE	International League Against Epilepsy
*KCNQ2*	Potassium voltage-gated channel subfamily Q member 2
MRI	Magnetic resonance imaging
NGS	Next-generation sequencing
VNS	Vagus nerve stimulator
VUS	Variants of uncertain significance
WES	Whole-exome sequencing
WGS	Whole-genome sequencing
