# Supplementary material for: Population-Based Study of Drug-Resistant Epilepsy Before Age Two: Predominance of Developmental and Epileptic Encephalopathies
Source: Neurol Int. 2026 Apr 22;18(5):76. doi: 10.3390/neurolint18050076 (PMC13209794; doi:10.3390/neurolint18050076)
Supplement: Supplementary file 1 [file neurolint-18-00076-s001.zip › neurolint-4221448-supplementary.pdf]

Table S1. A comprehensive list of all sequence variants, and the chromosomal aberration.

Pathogenic sequence variants														
**Pt.**	**Gene**	**Transcript**	**c.**	**p.**	**variant type**	**Automated ACMG classification (Varsome)**		**Manually revised ACMG classification**	**CADD**	**REVEL**	**Zygosity**	**HGVS**	**Assay**	**Comments**
1	*CDKL5*	NM_003159.3	c.2225_2228del	p.(Glu742Alafs*41)	frameshift	P	10P-0B	P	-	-	hemi (*de novo*)	NM_003159.3(CDKL5): c.2225_2228del p.(Glu742Alafs*41)	Panel	DOI: 10.1055/s-0036-1586730
2	*COL4A1*	NM_001845.6	c.4022-2del	p.?	splice site	LP	9P-0B	P	-	-	het (mat)	NM_001845.6(COL4A1):c.4022-2del p.?	Panel	
3	*DNM1*	NM_004408.4	c.1197-8G>A	p.?	non coding	LB	1P-2B	P		-	het (*de novo*)	NM_004408.4(DNM1):c.1197-8G>A p.?	Exome	affects splicing
4	*GABRG2*	NM_198904.4	c.964G>A	p.Ala322Thr	missense	VUS	4P-0B	P	25.7	0.647	het (*de novo*)	NM_198904.4(GABRG2):c.964G>A p.(Ala322Thr)	Panel	
5	*IRF2BPL*	NM_024496.4	c.240_243del	p.(Val81Profs*70)	frameshift	P	10P-0B	P	-	-	het (*de novo*)	NM_024496.4(IRF2BPL):c.240_243del p.(Val81Profs*70)	Genome	
6	*KCNQ2*	NM_172107.4	c.1762A>C	p.(Arg588=)	synonymous	VUS	5P-0B	P	22	-	het (*de novo*)	NM_172107.4(KCNQ2): c.1762A>C p.(Arg588=)	Exome	likely affects splicing
7	*KMT2D*	NM_003482.4	c.2579del	p.(Leu860Argfs*70)	frameshift	P	11P-0B	P	-	-	het (*de novo*)	NM_003482.4(KMT2D): c.2579del p.(Leu860Argfs*70)	Single gene testing	
8	*PCDH19*	NM_001184880.2	c.2057del	p.(Gly686Alafs*9)	frameshift	LP	9P-0B	P	-	-	het (*de novo*)	NM_001184880.2(PCDH19):c.2057del (p.Gly686Alafs*9)	Panel	
9	*PCDH19*	NM_020766.3	c.1091del	p.(Pro364Argfs*4)	frameshift	P	13P-0B	P	-	-	het (*de novo*)	NM_020766.3(PCDH19):c.1091del p.(Pro364Argfs*4)	Panel	
10	*PRRT2*	NM_145239.3	c.649dup	p.(Arg217Profs*8)	frameshift	P	17P-0B	P	-	-	het	NM_145239.3(PRRT2):c.649dup p.(Arg217Profs*8)	Panel	
11	*SCN1A*	NM_001165963.4	c.2837G>A	p.(Arg946His)	missense	P	21P-0B	P	28.1	0.987	het (*de novo*)	NM_001165963.4(SCN1A):c.2837G>A p.(Arg946His)	Panel	
12	*SYNGAP1*	NM_006772.3	c.3253del	p.(Arg1085Glyfs*45)	frameshift	LP	9P-0B	P	-	-	het (*de novo*)	NM_006772.3(SYNGAP1):c.3253del p.(Arg1085Glyfs*45)	Exome	
			c.3255del	p.(Pro1086Hisfs*44)	frameshift	LP	9P-0B	P	-	-	het (*de novo*)	NM_006772.3(SYNGAP1):c.3255del p.(Pro1086Hisfs*44)	Exome	

13	*TSC2*	NM_000548.5	c.3203C>T	p.(Thr1068Ile)	missense	P	17P-0B	P	24.4	0.932	het (*de novo*)	NM_000548.5(TSC2):c.3203C>T p.(Thr1068Ile)	Panel	
**Variants with unknown significance**														
14	*SCN2A*	NM_021007.3	c.1112G>A	p.(Ser371Asn)	missense	VUS	4P-0B	VUS	26.3	0.742	het (mat)	NM_021007.3(SCN2A): c.1112G>A p.(Ser371Asn)	Panel	
15	*SLC9A6*	NM_006359.3	c.472C>T	p.(Pro158Ser)	missense	VUS	3P-1B	VUS	24.4	0.648	hemi (mat)	NM_006359.3(SLC9A6): c.472C>T p.(Pro158Ser)	Panel	brother without epilepsy has the same variant
16	*SPTAN1*	NM_001130438.3	c.6422T>G	p.(Phe2141Cys)	missense	VUS	5P-1B	VUS	32	0.93	het	NM_001130438.3(SPTAN1):c.6422T>G p.(Phe2141Cys)	Panel	not inherited from mother, father unavailable for testing
17	*WNK3*	NM_020922.5	c.4996C>T	p.(Leu1666Phe)	missense	LB	1P-3B	VUS	24.2	0.053	hemi (mat)	NM_020922.5(WNK3):c.4996C>T p.(Leu1666Phe)	Exome	
**Novel disease gene candidates**														
18	*ACSL5*	NM_016234.4	c.1853A>G	p.(Tyr618Cys)	missense	VUS	5P-0B	GUS	27.3	0.694	c.het (pat)	NM_016234.4(ACSL5):c.1853A>G p.(Tyr618Cys)	Genome	
			c.*760A>G	p.?	non coding, 3'UTR	LB	1P-4B	GUS	9.9	-	c.het (mat)	NM_016234.4(ACSL5):c.*760A>G p.?	Genome	
	RNU2-2	NR_199791.1	n.35A>G		non coding			P	10.9	-	het (*de novo*)	NR_199791.1(RNU2-2):n.35A>G	Genome	DOI: https://doi.org/10.1038/s41588-025-02159-5
19	*DSCAM*	NM_001389.5	c.5116G>A	p.(Val1706Ile)	missense	VUS	2P-1B	GUS	23.3	0.115	het (*de novo*)	NM_001389.5(DSCAM): c.5116G>A p.(Val1706Ile)	Exome	
	LMTK3	NM_001388485.1	c.2190dup	p.(Glu731Argfs*809)	frameshift	VUS	4P-4B	GUS	-	-	het (*de novo*)	NM_001388485.1(LMTK3):c.2190dup p.(Glu731Argfs*809)	Exome	
20	*SIRT6*	NM_016539.4	c.701G>A	p.(Gly234Asp)	missense	VUS	1P-0B	GUS	26.1	-	c.het (pat)	NM_016539.4(SIRT6):c.701G>A p.(Gly234Asp)	Genome	patient 20 and 21 are siblings (triplets). Functional studies in process

			c.615-8G>A	p.?	non coding	VUS	5P-0B	GUS	20.9	-	c.het (mat)	NM_016539.4(SIRT6):c.615-8G>A p.?	Genome	splice-site, functional studies in process
21	*SIRT6*	NM_016539.4	c.701G>A	p.(Gly234Asp)	missense	VUS	1P-0B	GUS	26.1	-	c.het (pat)	NM_016539.4(SIRT6):c.701G>A p.(Gly234Asp)	Genome	patient 20 and 21 are siblings (triplets). Functional studies in process
			c.615-8G>A	p.?	non coding	VUS	5P-0B	GUS	20.9	-	c.het (mat)	NM_016539.4(SIRT6):c.615-8G>A p.?	Genome	splice-site, functional studies in process

Abbreviations: Pt.- patient, B - benign, LB - likely benign, VUS - variant of uncertain significance, LP - likely pathogenic, P - pathogenic, GUS - gene of uncertain significance, het - heterozygous, c.het - compound heterozygous, hom - homozygous, hemi - hemizygous, mat - maternal, pat - paternal.

Chromosomal aberrations

Patient	ISCN	Automated ACMG classification (CNV-ClinViewer)	Manually revised ACMG classification	Assay	Comments
22	46,XY,r(14)(p12q32)	-	P	karyotype	*de novo*
